# The Use of an Interactive Social Simulation Tool for Adults Who Stutter: A Pilot Study

**DOI:** 10.3390/ejihpe13010014

**Published:** 2023-01-13

**Authors:** Grant Meredith, Leigh Achterbosch, Blake Peck, Daniel Terry, Evan Dekker, Ann Packman

**Affiliations:** 1 Global Professional School, Federation University, Mt. Helen, Ballarat, VIC 3350, Australia; 2 Institute of Innovation, Science and Sustainability, Federation University, Mt. Helen, Ballarat, VIC 3350, Australia; 3 Institute of Health and Wellbeing, Federation University, Mt. Helen, Ballarat, VIC 3350, Australia; 4 The Australian Stuttering Research Centre, University of Technology Sydney, Sydney, NSW 2007, Australia

**Keywords:** stuttering, adults, simulation

## Abstract

This study reports a user evaluation of a DVD-based social simulator, developed for people who stutter to potentially gain confidence in using a learned fluency technique. The aim was to examine and evaluate the pilot of the DVD-based social simulator, Scenari-Aid, to inform the development of an online version of the program. Thirty-seven adults who were stuttering were recruited to the study from non-professional groups in Australia. The DVD comprised scenarios with actors in real-life settings that were designed to elicit verbal responses. Participants worked through the scenarios at their own rate and then completed an online survey. The survey comprised 29 statements requiring responses on a 5-point Likert scale and provided information about users’ perceptions of participating in the social simulations. There was high positive agreement among the participants on all statements, the most important being that they perceived the scenarios represented in everyday speaking situations and that they felt immersed in them. Participants also agreed that both their fluency and confidence increased in everyday speaking situations as a result of working through the DVD scenarios. The developers were satisfied that, despite the subjective nature of the findings, the study provided sufficient support for constructing the online version, which is now available to the public free of charge. Further research is needed to provide empirical evidence of the contribution it can make to the efficacy of speech programs for adults who stutter.

## 1. Introduction

### 1.1. Stuttering

Developmental stuttering impacts an estimated 1% of the global population. It typically starts in early childhood but while most children recover within the first few years, others continue to stutter throughout adolescence and adulthood. Despite decades of research, the cause of stuttering is not fully understood, although numerous theoretical causal explanations have been developed [1]. There is clear evidence emerging over many years, however, that people of all ages who stutter have reduced connectivity in those areas of the brain underpinning spoken language [2]. This, it has been proposed, renders the production of spoken language liable to disruption by certain linguistic task demands [3]. Genetics plays a part in stuttering and there is recent evidence of compromised white connective tissue in neonates who are genetically at risk of stuttering [4].

Stuttering involves involuntary disruptions of the flow of speech in the form of repeated sounds and syllabic units, and/or fixed postures of the speech organs that result in audible and/or inaudible prolongation of speech sounds [5]. These are frequently accompanied by verbal and nonverbal signs of struggle, including facial grimacing. Stuttering does not occur on every word and can vary between individuals in terms of the severity and frequency of stuttering moments, the latter ranging from 1% syllables stuttered (%SS) up to around 30%SS in some individuals. It can also vary within individuals, depending on the communicative context [3], increasing for many when anxious.

Stuttering interferes with effective verbal communication, even during the pre-school years [6]. During the school years, children who stutter typically avoid participating in classroom activities and are at increased risk of being teased and bullied [7]. Understandably, this typically leads to fear of being judged negatively by peers and hence to the development of social anxiety disorder [8,9]. For people who stutter, social anxiety disorder typically persists through adolescence and adulthood with a prevalence of 30–60% in adults [10]. It involves the individual’s negative thoughts and beliefs about how people will react to their stuttering and loss of confidence in the ability to participate effectively in situations requiring verbal interaction. They may develop complex avoidance strategies and coping mechanisms to hide or minimise their stuttering [11], such as avoiding certain words and changing their use of language to discourage dynamic interaction with a conversational partner [12]. It is not surprising stuttering can impact overall life satisfaction [13] and can limit an individual’s chances to achieve their educational and vocational potential [14,15].

### 1.2. Treatments for Adults Who Stutter

#### 1.2.1. Speech Treatment

According to a recent review [16], the speech treatment for adults who stutter with the highest empirical support involves modifying speech production in ways that are known to reduce the frequency and/or severity of stuttering, such as prolonging speech sounds. This is commonly referred to as speech restructuring (also known as smooth speech, or prolonged speech). Two well-supported speech restructuring programs are the Camperdown Program [17] and the Comprehensive Stuttering Program [18]. Participants learn the new speech pattern at a slow speech rate, increasing the rate gradually until speech sounds as natural as possible. The goal of the treatment is for participants to use this fluency technique in everyday speaking situations so they can speak with little or no stuttering. Generalising the use of the fluency technique in everyday speaking situations is essential, with feedback about its use given by the speech pathologist.

Speech restructuring is not a cure for stuttering, but simply gives the person a tool with which to control their stuttering. However, continuing to use a fluency technique effectively in everyday speaking situations is not easy, and typically requires ongoing practice, with support from a community group such as the Australian Speak Easy Association. Many adults continue to seek further speech pathology services in the form of maintenance groups and re-enrolment in a program, which can overload speech pathology services and be costly for individuals [19].

One reason for a failure to maintain the benefits of speech restructuring programs is the presence of social anxiety [20]. Iverach et al. [20] followed a group of 64 adults who participated in a speech-restructuring program and found that, after 6 months, the only participants who maintained the benefits of the program in everyday situations were the third who had no mental health disorders. Hence, reducing social anxiety would seem to be critical if speech-restructuring interventions are to be effective.

#### 1.2.2. Treatment for Anxiety

While anxiety about speaking has long been addressed in speech treatments for stuttering, there has been more recent interest in the use of psychological programs such as Acceptance and Commitment Therapy [21] and Cognitive Behaviour Therapy (CBT) [22]. The CBT program reported by Menzies et al. [22] was developed specially for people who stutter and have social anxiety. This program challenges negative thoughts and beliefs about stuttering while encouraging participants to enter feared situations and communicate freely. This program (iGlebe) is now available online on the website of the Australian Stuttering Research Centre (www.uts.edu.au/asrc/resources/iglebe, accessed on 08 January 2023).

#### 1.2.3. Issues Arising with Speech and Anxiety Treatments

Interestingly, there is some conflict between treatments such as speech restructuring and CBT [23]. According to Lowe et al. [23], the use of a fluency-inducing speech pattern can be seen as a safety behaviour. Safety behaviours, such as avoiding eye contact, are well-recognised in the field of psychology, and are used by socially anxious people to reduce immediate anxiety in social situations. This is often why people use a fluency technique; that is, it is used to reduce the speaker’s anxiety caused by perceived negative reactions of others during conversational interactions. However, it is well established in the field of psychology that while safety behaviours may serve to reduce anxiety during social interaction, they do not reduce the expectation of anxiety in future such interactions.

In addition, as discussed by Lowe et al. [23], the use of a fluency technique in communication contexts involves extreme self-focus. Constant self-directed kinaesthetic and auditory attention is required to manage the ongoing changes to speech motor control required to produce the learned modified speech pattern, while the speaker is concurrently formulating linguistic content. However, self-focus is discouraged in CBT, as it is also known to work against reducing anxiety. Lowe et al. [23] conclude that the two treatments for stuttering—one for fluency and the other for social anxiety—may at times, work against each other. For many people who stutter, however, there are situations such as the workplace and/or on the telephone in which minimal stuttering will be desirable. As Lowe et al. [23] conclude, the solution to this dilemma is not straightforward and further research is required.

### 1.3. Technology and Stuttering Treatment

In Australia, there are long waiting lists to access publicly funded speech pathology services, and once therapy has been accessed and established, only limited supported funding schemes are available to provide the long-term maintenance of their benefits [24]. There is also a lack of qualified practitioners in rural and remote areas [25].

Packman and Meredith [26] called for the greater use of technology in the management of stuttering such as web-based treatment and the use of virtual reality. This, it was argued, would increase access to treatments. Since that time, the use of telehealth has increased rapidly. For example, evidence-based speech and anxiety treatment programs for stuttering are being delivered by webcam and as standalone internet-based programs [10,27,28].

#### The Use of Social Simulation

As stated, a critical part of speech programs like speech restructuring is that participants use their fluency technique in the real world. CBT also involves participants engaging in the real world to improve information processing. It would seem, then, that the use of social simulation could be a useful adjunct to these treatments. The use of role-playing and social simulations are well-established practices in a range of health-related disciplines both for clinical training and for improving patient experience [29,30]. In real-world simulations, people physically play out different scenarios without having to enter real-life situations. In terms of stuttering, the social simulation would seem to make an important contribution to treatments, regardless of whether it is delivered face-to-face in a clinic, by webcam, as virtual reality (VR), or as a standalone internet version. If freely available, it would seem social simulation could enhance stuttering treatments for those with limited access to services.

One example of social simulation used with adults who stutter is virtual reality [31]. Brundage and colleagues [32,33] have used VR as a way of assessing—within the clinic—the generalization of speech treatment effects beyond the clinical setting. In a VR experience (VRE), the moving visual stimulus is presented via a head-mounted display [32]. Brundage et al. [32] developed two job interview VREs and found that stuttering increased significantly for 23 adults during the challenging interview as compared to the supportive interview. Brundage and Hancock [33] had 10 adults who were stuttering give a presentation to a live audience and to a VRE-simulated audience. Assessments based on reports of confidence as a speaker and apprehension about speaking, completed just prior to each presentation, were similar for both the live and the virtual audiences. The authors reported that the findings of the two studies indicate the similarity between VREs and experiences in the real world and hence, that VR may be of value in the assessment and treatment of stuttering.

A social simulation program that has been developed led by the first two authors of this paper for people who stutter and is freely available on the internet is Scenari-Aid (www.scenariaid.com, accessed on 08 January 2023). It does not use VR but is a web-based series of simulated social scenarios. The aims of the social simulations in Scenari-Aid are to (1) assist people who stutter to practice fluency techniques, (2) assist users over time to alleviate general levels of speech-related anxiety, (3) boost associated social skills, and (4) build confidence in transferring fluency skills into real-world interactions. There are eight categories of simulated situations with numerous examples in each category. The video clips were recorded with actors, in real-life settings, asking such questions as “What can I get for you today?” Scenari-Aid is available to the public free of charge.

### 1.4. The Present Study

The present study reports the development of Scenari-Aid, conducted prior to it being uploaded to the internet for public use. A DVD-delivery platform was used, which is henceforth referred to as Scenari-Aid-DVD.

Scenari-Aid-DVD comprised 25 scenarios replicating real-life situations. As with the current online Scenari-Aid, actors were filmed in real-life settings, portraying interactive social situations that adults who stutter frequently report finding difficult. These categories were formed by the lead author after discussions with people who stutter via associated conferences and social media groups. There were 25 scenarios in the following seven categories:

1. Job interviews (solo and panel interviews);
2. Medical (visiting a general practitioner);
3. Cafes and restaurants (ordering light food and refreshments);
4. Fast food (ordering convenient foods);
5. General shops (expressing needs, such as in a bookshop, hairdresser, butcher);
6. Theatre (ordering tickets and snacks);
7. Appliance stores (discussing buying a television, microwave, or refrigerator).

Each category contained video clips presented in an ordered lineal narrative order, mirroring a staged real-life series of targeted verbal interactions. There were 91 video clips in all. The following clips are part of a job interview scenario:

- Clip #1: “Why do you want to work for this organization?”
- Clip #2: “Tell me about some of your career highlights and in particular any experiences that relate specifically to this position you have applied for.”
- Clip #3: “What are your career goals? Where do you see yourself five or ten years from now?”
- Clip #4: “What new skills or ideas do you bring to this job that our internal candidates do not offer?”
- Clip #5: “What are your preferred working conditions, working alone or in a group and why?”

Scenari-Aid-DVD used a hierarchical navigational structure similar to a film DVD menu system and was designed to be intuitive to use. The end product, the current online Scenari-Aid, was conceptualised primarily as a way for adults who stutter to practice their fluency techniques. The developers wanted to know if this was achieved with Scenari-Aid-DVD and whether confidence in real-life speaking situations improved with use over time.

Overall, the aim was to examine and evaluate the pilot of the DVD-based social simulator, Scenari-Aid, to inform the development of an online version of the program.

## 2. Materials and Methods

### 2.1. Participants

Thirty-seven adults who were stuttering participated in the study, comprising 11 females, (30%) and 26 males (70%), which is similar to the adult female-to-male ratio in the community of around one female to every four males [34]. The age groups of participants were more evenly spread: 18–25 years (*n* = 12, 32%), 26–35 years (*n* = 10, 27%), 36–45 years (*n* = 11, 30%), and older than 45 years (*n* = 4, 11%), as was their self-rated level of stuttering, with almost half (*n* = 17, 46%) identifying themselves as moderate stutterers.

Recruitment was made via contact with two non-professional Australian stuttering organisations. Information about the Scenari-Aid-DVD study and an application form were supplied to each organisation, and these were distributed to their member base. Members were informed there was no cost to participants and were instructed to contact the first author if they were interested in participating in the study. The first author then mailed interested persons the Scenari-Aid-DVD, a Plain Language Information Statement (PLIS) regarding the study, and the survey. It must be noted that some participants had early access to the Scenari-Aid-DVD due to the first author making it freely available to non-professional Australian stuttering organisations pre to this study. Users were instructed to work through the scenarios in their own time and fashion, and to complete and return the survey to the first author if and when they felt confident and comfortable enough to provide feedback on their use of the DVD. Overall, 97 people indicated their further interest to participate and 37 (38%) chose to commit to the following survey.

### 2.2. Survey

After reviewing the Scenari-Aid-DVD and agreeing to complete the feedback survey, participants were directed to read the PLIS and to provide their informed consent. After providing written consent, participants were then directed to an online survey, which contained 29 questions exploring their perceptions of Scenari-Aid-DVD. Key elements of the survey explored the DVD programme’s usability and participant engagement, its value to therapy and fluency techniques, its impact on social anxiety, and its general value as a product for people who stutter. The questions were developed by the multi-disciplinary research team to cover aspects of product design, user experience, and perception of impact upon fluency and social speech confidence.

The survey comprised Likert-scaled statements each of which had a 5-point range for response options: Strongly Agree = SA; Agree = A; Neither Agree nor Disagree = N; Disagree = D; Strongly Disagree = SD. The study was approved by the Federation University Australia Human Research Ethics Committee (B11-014).

The survey remained open for a period of eight months and was completed by 37 respondents. All interested individuals who were sent a copy of the Scenari-Aid DVD completed the survey.

### 2.3. Data Analysis

Data were cleaned, checked, and analysed using Microsoft Excel. Data were then collated and grouped by question thematically as shown in the following tables. Descriptive summary statistics were used, including frequencies and percentages to provide an overall account of the results. This was chosen due to the small number of participants, and because it was a technology pilot evaluation that would inform a much larger project which will go through more a rigorous evaluation; see Section 4.1 for further details.

## 3. Results

### 3.1. Survey Findings

Most respondents had used Scenari-Aid for a duration of six to eight months (*n* = 16, 43%) or more than ten months (*n* = 15, 41%) indicating that the majority had been using Scenari-Aid-DVD from a time before the survey was deployed. As described earlier in this article, this is because some participants had early access to Scenari-Aid due to it being already available to the members of the two participating non-professional Australian stuttering organisations. All participants had been using Scenari-Aid at home, with 13 (35%) using it at their place of work.

There were three categories of statements requiring responses, *Perceptions of Fluency* (nine statements), *Value to Therapy and Fluency Techniques* (13 statements), and *Perceptions of Anxiety* (seven statements). To assess the extent of agreement within each category, the SA and the A responses were combined for positive statements and the D and SD responses for the negative statements. This provided the extent of positive responses to each of the 29 statements.

#### 3.1.1. Perceptions of Fluency

The results for the nine statements in this category are shown in Table 1.

Positive responses (see above) to each of the nine statements ranged from 84–97%, with seven of these 90% or over. The highest agreement (97%) was for statements 3, 5, 8 and 9. This indicates reported improvements in confidence and the acquisition of effective communication skills (3,8) and recommends others who stutter to use Scenari-Aid-DVD (5,9). In three of the nine statements, a few participants perceived that the software had not improved their fluency.

#### 3.1.2. Value to Therapy and Fluency Techniques

The results for the 13 statements about the perceived value of Scenario-Aid-DVD, in terms of complementing therapy and assisting in the use of speech techniques, are shown in Table 2.

There was slightly less agreement (range: 76–97%) for these 13 statements; however, there was over 90% agreement for seven of them (2,3,4,7,8,11,13). These responses suggested that the scenarios were challenging to work through at first (3), but that the DVD gave a better understanding of how to manage one’s speech (8, 11) and to practice when needed (2), that the DVD improved confidence in using skills in real life situations (11,13), and that Scenari-Aid-DVD should be introduced into therapy programs (4) and used in support group environments (7).

#### 3.1.3. Perceptions of Anxiety

The results for the seven statements about participants’ perceptions of how Scenari-Aid has assisted them with social anxiety and fears are shown in Table 3.

Agreement for all seven statements was extremely high (range 97–100%,) with 100% agreement for four statements (2, 5, 6, 7). This level of agreement indicated that the DVD provided situations that respondents found anxiety-provoking and that working through them eased levels of anxiety when facing them in real life.

## 4. Discussion

The aim of this study was to provide preliminary evidence to support the development of the current online Scenari-Aid (www.scenariaid.com, accessed on 08 January 2023). This social simulator provides real-life videos of interactive situations prompting verbal responses. Scenari-Aid-DVD, which was used in the present study, had examples of such scenarios, and participants were invited to complete a survey on their user experience. All participants were adults who stutter

A critical finding of the survey is that all participants indicated that the social interaction scenarios in Scenari-Aid-DVD mirrored real-life situations, with all but one participant reporting that these were feared and stressful situations for them. Many participants reported that using Scenari-Aid-DVD assisted them to manage their speech and enabled them to reflect critically on their related progress. Scenari-Aid-DVD also appeared to be valued in terms of offering long-term support by way of participants wanting to continue using the product.

Importantly, all but two participants reported that the Scenari-Aid-DVD provided opportunities to practice their more general communication skills, and the confidence that this provided transferred into real-life situations. All but one participant indicated that using Scenari-Aid-DVD regularly helped to ease their apprehensions about challenging themselves to face social situations that perhaps in some cases they felt out of their comfort zone. Importantly, all but one participant agreed that they would recommend the Scenari-Aid-DVD to other people who stutter. The finding that the use of Scenari-Aid-DVD appears to be easing social apprehensions concerning the transference of speaking skills into the real world is a positive outcome. It suggests that the platform could be considered by speech professionals to complement existing speech restructuring programs by helping to address the concerns raised by Iverach and colleagues [20] about how untreated social anxiety may impact the success of speech restructuring programs. However, online Scenari-Aid should not be considered a substitute for CBT.

These results for video-produced lineal roleplaying scenarios of Scenari-Aid-DVD and their applicability were encouraging. It was decided that they provided sufficient evidence for the development of the online Scenari-Aid social simulator, with the similar aim of supporting the needs of adults who stutter and want to practise speaking in challenging speaking situations. As a result, the Scenari-Aid website was created with a larger scenario set (106 total) containing over 600 individual clips and covering a larger range of scenarios that had been suggested by participants and non-studied users. The new scenarios include public transport, social discussions, and more in-depth medical situations. To date, with no promotional campaigns, the website has attracted close to 5000 Australian and International-registered individual and clinically based users.

As mentioned, an important finding is participants’ reports of experiencing a strong sense of immersion when participating in the Scenari-Aid-DVD scenarios, something that has been previously reported for more technologically sophisticated software such as virtual reality [32,33]. Although yet to be assessed, this sense of immersion appears to also be generated by the online Scenari-Aid. Interestingly, Scenari-Aid not only provides scenarios for users who stutter to practice fluency techniques but also provides opportunities for them to work on the pragmatics of their social interactions; that is, to develop an appropriate use of language according to the situations and conversational partners engaged in. The ability to pause and reflect during a simulation enabled a user to approach speaking situations without the stress of a listener expecting an instant response. The Scenari-Aid-DVD also gives the user the opportunity to recognise and work on, what Lowe et al. [23] describe as safety behaviours, in order to better manage both their anxiety levels and fluency levels once transferred into real-world settings. From this perspective, Scenari-Aid may also be helpful for others wanting to improve the communications skills, such as adults with aphasia and those learning English as a second language.

### 4.1. Limitations

The small sample size of participants is a limitation, although it was considered acceptable for this pilot study, the aim of which was to gather user perceptions. Research suggests that 30 [35] and 35–40 [36] participants for a pilot study provide data reliability, whereas smaller pilot studies of 10–15 participants can sometimes be imprecise for preliminary data [35]. Additionally, although only a few questions addressed usability, it has been found in usability testing that with at least 20 participants, 90% to 95% of usability problems will be revealed [37,38]. Therefore, the developers decided that the perceptions of the 37 participants were sufficient for the pilot and to justify the development of the online product.

Although the demographic of the participants is considered representative in terms of age, it is possible that the sample who self-selected to be part of the study may have had higher levels of confidence and as a result may have achieved higher levels of success from the programme.

Another possible limitation of the study is that most statements were positive and may have elicited positive responses from the participants. However, the use of Likert scales would have reduced that likelihood.

A further limitation is the lack of formal assessments of participant stuttering severity, confidence using their fluency technique, and social anxiety levels. This should be investigated in future research.

### 4.2. Future Research

Future directions for research would include clinical trials of the lineal scenario premise with people who stutter. Clinical trials would be important to validate the contribution of products such as Scenari-Aid to conventional stuttering programs, with professional ratings of participants’ stuttering and anxiety levels pre- and post-trial in addition to participant perceptions. Predicting which participants benefit from social simulation would be critical.

## 5. Conclusions

The Scenari-Aid DVD was framed as a pilot study for developing an online and expanded version of Scenari-Aid. Importantly, free-to-use Scenari-Aid provides a means of addressing the lack of access to health services in rural Australia and indeed in many other countries around the world that have access to the internet. This pilot study has supported the ideal raised by Packman and Meredith [28] that greater use of technology in the management of stuttering needs to be trailed and evaluated in the future.

## Figures and Tables

**Table 1:** Perceptions of Fluency.

	Answer				
Question	SA	A	N	D	SD
1. As I continued through the scenarios, I found my fluency improving.	7	28	1	1	
2. As I continued working through the scenarios, I did not find my speech improving.	0	1	1	23	12
3. As I worked through the scenarios, I found my confidence in speech improving.	10	26	1	0	0
4. Using Scenari-Aid has strengthened my overall level of fluency.	9	23	5	0	0
5. I would suggest to other people who stutter to use Scenari-Aid as a tool to aid with their fluency training.	14	22	1	0	0
6. Using Scenari-Aid has assisted me in embracing my speech techniques.	6	27	4	0	0
7. Scenari-Aid has not helped me to prepare for real-life speaking situations.	1	1	1	24	10
8. Using Scenari-Aid has assisted me in developing the skills I require to communicate effectively.	5	31	1	0	0
9. I would suggest to other people who stutter to use Scenari-Aid as an aid with gaining social speaking confidence.	14	22	1	0	0

**Table 2:** Value to Therapy and Fluency Techniques.

	Answer				
Question	SA	A	N	D	SD
1. Scenari-Aid offered me the flexibility of location to practise.	8	20	8	1	0
2. Scenari-Aid allowed me to practise at times of need.	12	22	2	1	0
3. At first, I found working through the scenarios challenging speech-wise.	9	27	0	1	0
4. I would not recommend Scenari-Aid being introduced into therapy-based programs for people who stutter.	1	0	1	20	15
5. Scenari-aid complemented my existing speech practice regime.	5	25	6	0	1
6. Scenari-Aid has not assisted me with transferring my speech techniques into the real world.	1	2	1	23	10
7. I would feel comfortable using Scenari-Aid within a support group environment.	15	20	1	1	0
8. Using Scenari-Aid has given me a better understanding of how better to manage my speech.	4	30	3	0	0
9. Using Scenari-Aid has assisted me to critically view how my speech is progressing.	8	23	5	1	0
10. Scenari-Aid has not helped me to develop a better understanding of how speech techniques could be used in a real-life situation.	0	2	2	27	6
11. I believe Scenari-Aid can assist me to further develop the required skills to confidently speak in the supplied situations.	9	26	2	0	0
12. I will continue to use Scenari-Aid to assist me to practise my speech.	6	27	4	0	0
13. I can apply my experiences of working through simulations to real-life scenarios.	10	25	2	0	0

**Table 3:** Perceptions of Anxiety.

	Answer				
Question	SA	A	N	D	SD
1. Scenarios presented representative feared situations.	13	23	1	0	0
2. Scenari-Aid presented me with challenging situations.	13	24	0	0	0
3. Working through the scenarios did not ease my apprehensions about facing them in real life.	0	0	1	26	10
4. Using Scenari-Aid has eased my levels of anxiety when approaching the presented scenarios in real life.	10	26	1	0	0
5. The scenarios provided covered situations that I have had trouble working through in real life.	12	25	0	0	0
6. The scenarios provided covered situations which at times cause me anxiety due to my stuttering.	14	23	0	0	0
7. The scenarios provided were ones with which I required more confidence in interacting.	11	26	0	0	0

## Data Availability

The data presented in this study are available on request from the corresponding author.
